# Reassortant Pandemic (H1N1) 2009 Virus in Pigs, United Kingdom

**DOI:** 10.3201/eid1706.101886

**Published:** 2011-06

**Authors:** Wendy A. Howard, Steve C. Essen, Benjamin W. Strugnell, Christine Russell, Laura Barrass, Scott M. Reid, Ian H. Brown

**Affiliations:** Author affiliations: Veterinary Laboratories Agency–Weybridge, Addlestone, UK (W.A. Howard, S.C. Essen, C. Russell, L. Barrass, S.M. Reid, I.H. Brown);; Veterinary Laboratories Agency–Thirsk, Thirsk, UK (B.W. Strugnell)

**Keywords:** Influenza, pandemic (H1N1) 2009 virus, viruses, pigs, reassortant viruses, United Kingdom, dispatch

## Abstract

Surveillance for influenza virus in pigs in the United Kingdom during spring 2010 detected a novel reassortant influenza virus. This virus had genes encoding internal proteins from pandemic (H1N1) 2009 virus and hemagglutinin and neuraminidase genes from swine influenza virus (H1N2). Our results demonstrate processes contributing to influenza virus heterogeneity.

During the 1918 influenza pandemic, the virus likely passed from humans to pigs (*1*). Descendants of this virus (classical swine influenza viruses), first isolated in 1930 (*2*), have continued to circulate in pigs (*1*). Other influenza viruses have caused either sporadic or enzootic infections.

Until 2009, the predominant influenza virus subtypes in pigs in Europe were avian-like (H1N1), human-like (H3N2) (representing virus transmissions from birds and humans, respectively), and H1N2 (*3*). Subtype H1N2 viruses, first identified in the United Kingdom in 1994 and subsequently detected throughout Europe, arose by reassortment between human subtype H1N1 (hemagglutinin [HA] gene), human-like swine subtype H3N2 (neuraminidase [NA] gene), and avian-like swine subtype H1N1 viruses (internal gene segments *4**,**5*; ).

Classical swine influenza viruses (H1N1) were dominant in North America (*6*). However, during the 1990s, infection of pigs with human subtype H3N2 virus resulted in viruses containing a triple-reassortant group of internal genes. These viruses contain genes derived from human, classical swine, and avian-origin viruses and can accept different HA and NA genes (*6*).

Pandemic (H1N1) 2009 virus is a reassortant virus with genes from recent North American triple reassortant (basic polymerase 2 [PB2], PB1, acidic polymerase, HA, nucleoprotein [NP], nonstructural gene) and European avian-like subtype H1N1 (NA, matrix [M]) viruses (*7*). Infections of domestic pigs with pandemic (H1N1) 2009 virus have been detected worldwide. In January 2010, a reassortant virus that contained a pandemic (H1N1) 2009 virus NA gene and an avian-like subtype H1N1 HA gene was detected in pigs in Hong Kong (*8*). This reassortant was efficiently transmitted between pigs (*8*). We report detection and characterization of a novel swine reassortant virus in the United Kingdom that has genes encoding internal proteins from pandemic (H1N1) 2009 virus and HA and NA genes from a swine subtype H1N2 virus.

## The Study

In mid-April 2010, influenza-like illness was reported in pigs in a North Yorkshire gilt (female pig intended for breeding that has not farrowed) grower unit of ≈1,200 animals. Gilts were brought into the unit in batches of ≈100 animals at ≈5 months of age. The first batch of gilts arrived in mid-January 2010; previously, the unit did not contain animals for >4 months. Gilts were housed in stable groups of ≈20 in a naturally vented building with a straw yard and remained in the unit for ≈70 days. The nearest pig farm was ≈3 miles away.

A persistent moist cough and signs typical of epizootic swine influenza were observed in 40%–50% of a batch of pigs 2 weeks after their arrival. Seven days after the onset of clinical signs, nasal swabs and serum samples were obtained from 6 pigs, and serum samples were obtained from 4 acutely affected pigs. Convalescent-phase serum samples were obtained from 9 pigs in the same batch 21 days later. Clinical signs had subsided by early June 2010.

Total RNA was extracted from swab eluant and amplified by using an M gene real-time reverse transcription PCR (RT-PCR) capable of detecting pandemic (H1N1) 2009 virus (*9*); 4 of 6 swabs were positive. None of the samples were positive for pandemic (H1N1) 2009 virus with a modified real-time RT-PCR specific for the HA gene (*9*). Only the sample positive by real-time RT-PCR with the lowest cycle threshold value yielded virus when inoculated into embryonated fowl eggs (*10*). Egg-grown virus was identified as subtype H1N2 by using hemagglutinin inhibition (HI) and NA inhibition with standard methods (*10*) and designated A/swine/England/1382/10 (H1N2). The virus was reisolated from the original sample to exclude cross-contamination.

A/swine/England/1382/10 was characterized by using whole genome sequencing and phylogenetic analysis. Gene fragments were amplified by using a 1-step real-time RT-PCR (QIAGEN, Hilden, Germany), and HA and NA genes were sequenced by using subtype H1N2 virus–specific primers (*5*). Partial internal gene segment sequencing was initially performed by using primer pairs (*5*). Full sequencing of internal gene segments used universal (NP, M, and nonstructural genes) and pandemic (H1N1) 2009 virus–specific primers (PB2, PB1, acidic polymerase, and NP genes). Primer sequences are available upon request.

Analysis of sequence data by BLAST analysis (http://blast.ncbi.nlm.nih.gov) determined the closest similarity to influenza virus isolates in the GenBank database. A/swine/England/1382/10 had HA and NA genes closely related to UK swine subtype H1N2 viruses (Table 1). All genes encoding internal proteins showed the highest similarity to pandemic (H1N1) 2009 viruses (Table 1).

**Table 1:** Genotypes of influenza virus subtype H1N2 and H1N1 isolates from pigs, United Kingdom, 2009–2010*

Virus	Date of sampling	Subtype	Gene segment							
			PB2	PB1	PA	HA	NP	NA	M	NS
A/swine/England/236/09	2009 Sep 28	H1N2	A	A	A	H	A	H	A	A
A/swine/England/1157/09	2009 Oct 5	H1N2	A	A	A	H	A	ND	A	A
A/swine/England/1428/09	2009 Dec 8	H1N2	A	A	A	H	A	H	A	A
A/swine/England/523/10	2010 Jan 4	H1N2	A	A	A	H	A	H	A	A
A/swine/England/1382/10†	2010 Apr 13	H1N2	P	P	P	H	P	H	P	P
A/swine/England/1389/10	2010 Apr 15	H1N1	A	A	A	A	A	A	A	A
A/swine/England/73690/10	2010 Jun 23	H1N1‡	P	P	P	P	P	P	P	P

*PB, basic polymerase; PA, acidic polymerase; HA, hemagglutinin; NP, nucleoprotein, NA, neuraminidase; M, matrix; NS, nonstructural; A, genes with closest homology to European avian-like swine influenza viruses (H1N1); H, genes with closest homology to European swine influenza viruses (H1N2); ND, gene not sequenced but typed as N2 by using standard NA inhibition assay; P, genes with closest homology to pandemic (H1N1) 2009 virus. Closest matching homologous genes were determined by analyzing nucleotide sequences (minimum of 180 bases) against the National Center for Biotechnology Information GenBank database (http://blast.ncbi.nlm.nih.gov). GenBank accession nos. JF290388–JF290395 and JF297995–JF298002. †A/swine/England/1382/10 reassortant virus. ‡Pandemic (H1N1) 2009 virus.

HA and NA genes of A/swine/England/1382/10 grouped within the European swine subtype H1N2 cluster, specifically, with contemporary subtype H1N2 isolates from England. The closest matching isolate for HA and NA was A/swine/England/1428/09, which is reported in this article (Figure).

**Figure:**
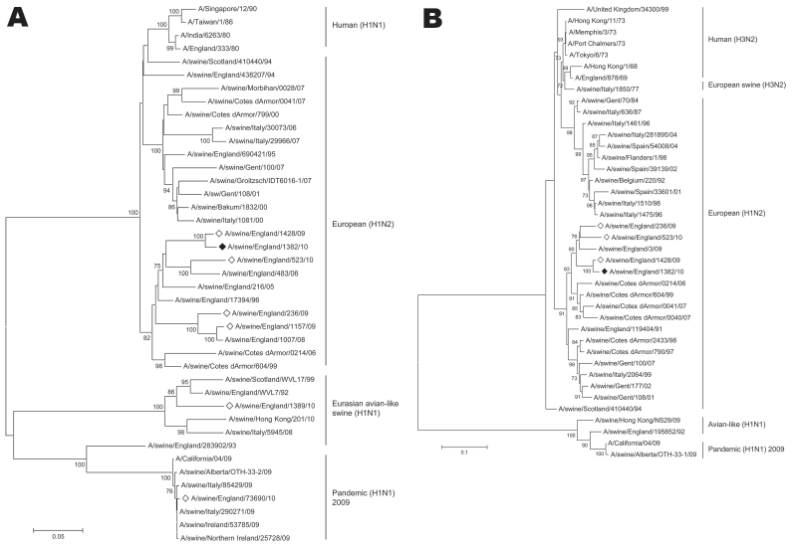
Phylogenetic analysis of influenza A virus hemagglutinin (A) and neuraminidase (B) genes. Trees were constructed by using the neighbor-joining method. Solid diamonds indicate A/swine/England/1382/10 genes from virus isolated in this study, and open diamonds indicate genes from other viruses reported in this study. Percentage of replicate trees in which the associated taxa clustered together in the bootstrap test (1,000 replicates) is shown next to the branches for values >70% (*11*). Evolutionary distances were computed by using the Tamura-Nei method (*12*). Phylogenetic analyses were conducted by using MEGA4 (*13*). Scale bars indicate nucleotide substitutions per site.

The M gene of A/swine/England/1382/10 had the S31N amantadine-resistance mutation, typical of pandemic (H1N1) 2009 viruses. It also had 627E and 701D mutations in the PB2 gene and mutation 591R, a basic amino acid that reportedly compensates for lack of the 627K mammalian-adaptive mutation (*14*). The PB1-F2 open-reading frame encoded a truncated PB1-F2 protein of 11 aa, consistent with other pandemic (H1N1) 2009 viruses. The NA gene has mutations 119E and 292R, which are associated with susceptibility to oseltamivir in N2 subtypes.

Since the emergence of pandemic (H1N1) 2009 virus, 5 other subtype H1N2 viruses have been detected in pigs in the United Kingdom. Partial sequencing of internal genes of these viruses showed they were not reassortants (Table 1). Reassortment was not detected in a European avian-like swine subtype H1N1 virus isolated from another pig unit in the same region in April 2010, or in a pandemic (H1N1) 2009 virus isolated from another pig unit in the same region in June 2010 (Table 1).

The 10 acute-phase and 9 convalescent-phase serum samples were subjected to standard HI tests (*10*) with antigens from A/swine/England/195852/92 (avian-like subtype H1N1), A/swine/England/1353/09 pandemic (H1N1) 2009 virus, A/swine/England/438207/94 (subtype H1N2), and homologous A/swine/England/1382/10 (Table 2). Acute-phase serum samples were positive for antibodies against pandemic (H1N1) 2009 virus. Titers increased >10-fold in convalescent-phase serum samples. Antibody titers to endemic and reassortant subtype H1N2 viruses were negligible in acute-phase serum samples but increased 14-fold and 16-fold, respectively, in convalescent-phase serum samples.

**Table 2:** Serologic cross-reactivity titers of acute-phase and convalescent-phase swine serum samples against subtypes of swine influenza viruses, United Kingdom*

Phase and pig no.	Virus			
	A/Swine/England/438207/94, subtype H1N2	A/Swine/England/195852/92, subtype H1N1	A/swine/England/1353/09, pandemic (H1N1) 2009	A/swine/England/1382/10, reassortant subtype H1N2
Acute phase				
1	<10	40	320	<10
2	<10	<10	160	<10
3	<10	<10	160	<10
4	<10	<10	320	<10
5	<10	<10	160	<10
6	<10	<10	320	<10
7	<10	<10	640	<10
8	<10	<10	320	<10
9	<10	<10	<10	<10
10	<10	<10	80	<10
Convalescent phase				
1	80	20	2,560	160
2	<10	<10	5,120	160
3	<10	<10	2,560	80
4	160	160	1,280	160
5	<10	<10	1,280	80
6	640	160	5,120	160
7	<10	<10	2,560	320
8	160	40	5,120	320
9	320	320	2,560	160

*Acute-phase serum samples were obtained from 10 pigs in the same batch during the time when clinical signs were apparent (≈2 wk after arrival at the unit). Convalescent-phase serum samples were obtained from 9 pigs in the same batch 21 d later. Standard hemagglutinin inhibition assays were conducted with subtype H1N1, pandemic (H1N1) 2009, and subtype H1N2 virus antigens derived from UK swine isolates. Homologous A/swine/England/1382/10 antigen was also used.

## Conclusions

We report detection of a novel reassortant virus between pandemic (H1N1) 2009 virus and a swine subtype H1N2 virus. In contrast to an earlier report of a reassortant virus that contained the NA gene of pandemic (H1N1) 2009 virus (*8*), in this study all genes encoding internal proteins of A/swine/England/1382/10 virus are derived from pandemic (H1N1) 2009 virus.

The source of A/swine/England/1382/10 could not be established. Appearance of clinical signs 2 weeks after arrival in the unit suggests that pigs were not previously infected with either a precursor or reassortant virus. However, detection of antibodies against pandemic (H1N1) 2009 virus in pigs coinciding with appearance of clinical signs suggests earlier subclinical infection with pandemic (H1N1) 2009 virus preceding co-circulation of subtype H1N2 or reassortant H1N2 viruses once pigs arrived at the unit. Earlier sampling of pigs in the unit may have detected subclinical precursor viruses.

We did not find evidence of similar reassortants in the United Kingdom. Therefore, it is unclear whether A/swine/England/1382/10 can be transmitted between pigs or has any selective advantage.

Our serologic results and those of others (*15*) indicate that antibodies against pandemic (H1N1) 2009 virus or subtype H1N2 virus produced during natural infection of pigs do not show cross-reactivity in HI tests. Therefore, pandemic (H1N1) virus and subtype H1N2 virus may continuously circulate in pigs in Europe, providing additional opportunities for reassortment.
